# Poisons—Their Effects and Detections

**Published:** 1885-08

**Authors:** 


POISONS: THEIR EFFECTS AND DETECTION. A Manual for the Use of Analytical Chemists and Experts, with an Introductory Essay on the Growth of Modern Toxicology. By Alexander Wynter Blyth, M. R. C. S., F. C. S., etc., Public Analyst for the County of Devon, and Medical Officer of Health and Public Analyst for St. Marylebone, with tables and illustrations. Volume I. New York: William Wood & Co., 56 and 58 Lafayette Place. 1885.

While thirty-three pages of this book at the opening are taken up with contents, there is no alphabetic index at the close to facilitate reference to the various matters of importance treated of in the 333 pages that make up this first volume, and it is to be hoped that such a table will be affixed at the close of the work.

The liability to accidental poisoning by mistakes in filling prescriptions, the suicidal tendencies of the age, and the occasional malicious use of various agents of this class with murderous intent, renders this work on poisons, with their antidotes, a most acceptable offering to the medical public.

The present volume completes an entirely re-written and greatly enlarged second edition of the author's "Practical Chemistry," and whenever possible, "natural" groups of poisons have been formed.

